# Withdrawing intra-aortic balloon pump support paradoxically improves microvascular flow

**DOI:** 10.1186/cc9242

**Published:** 2010-08-25

**Authors:** Luuk DH Munsterman, Paul WG Elbers, Alaattin Ozdemir, Eric PA van Dongen, Mat van Iterson, Can Ince

**Affiliations:** 1 Department of Translational Physiology, Academic Medical Center, University of Amsterdam, Amsterdam, PO box 22.660 1100DD, The Netherlands; 2 Department of Anesthesiology, Intensive Care and Pain Medicine, St. Antonius Hospital, Nieuwegein, PO box 2500 3430EM, The Netherlands

## Abstract

**Introduction:**

The Intra-Aortic Balloon Pump (IABP) is frequently used to mechanically support the heart. There is evidence that IABP improves microvascular flow during cardiogenic shock but its influence on the human microcirculation in patients deemed ready for discontinuing IABP support has not yet been studied. Therefore we used sidestream dark field imaging (SDF) to test our hypothesis that human microcirculation remains unaltered with or without IABP support in patients clinically ready for discontinuation of mechanical support.

**Methods:**

We studied 15 ICU patients on IABP therapy. Measurements were performed after the clinical decision was made to remove the balloon catheter. We recorded global hemodynamic parameters and performed venous oximetry during maximal IABP support (1:1) and 10 minutes after temporarily stopping the IABP therapy. At both time points, we also recorded video clips of the sublingual microcirculation. From these we determined indices of microvascular perfusion including perfused vessel density (PVD) and microvascular flow index (MFI).

**Results:**

Ceasing IABP support lowered mean arterial pressure (74 ± 8 to 71 ± 10 mmHg; *P *= 0.048) and increased diastolic pressure (43 ± 10 to 53 ± 9 mmHg; *P *= 0.0002). However, at the level of the microcirculation we found an increase of PVD of small vessels <20 μm (5.47 ± 1.76 to 6.63 ± 1.90; *P *= 0.0039). PVD for vessels >20 μm and MFI for both small and large vessels were unaltered. During the procedure global oxygenation parameters (ScvO_2_/SvO_2_) remained unchanged.

**Conclusions:**

In patients deemed ready for discontinuing IABP support according to current practice, SDF imaging showed an increase of microcirculatory flow of small vessels after ceasing IABP therapy. This observation may indicate that IABP impairs microvascular perfusion in recovered patients, although this warrants confirmation.

## Introduction

In cardiogenic shock, intra-aortic balloon counterpulsation is frequently used to mechanically support the failing heart [1,2]. Intra-Aortic Balloon Pump- (IABP-) support improves coronary blood flow by augmenting systemic and coronary diastolic blood pressure and increases cardiac index by reducing left ventricular work [1,3]. As a bridge to recovery, its goal is to facilitate the heart and continuously provide adequate systemic perfusion.

However, the microcirculation is ultimately responsible for delivering oxygen and substrates to tissue [4]. The recent emergence of Orthogonal Polarization Spectral imaging and its successor sidestream dark field imaging (SDF) has enabled imaging of the human microcirculation in real time [5-7]. These techniques have been used to characterize the microcirculation in various clinical situations including cardiogenic shock.

For example, De Backer *et al. *[8] demonstrated that microvascular blood flow worsens in severe cardiac failure and cardiogenic shock and is associated with in-hospital mortality. Importantly, there is now a large body of evidence that microvascular flow may be relatively independent from global hemodynamics [4]. For example, arterial and venous blood pressure, cardiac output as well as central or mixed venous oxygen saturation may not necessarily reflect microvascular perfusion [9-14]. However, in current clinical practice, these very global hemodynamic parameters frequently guide the decision when to start and withdraw IABP-therapy.

Three recent trials examined the microcirculatory effects of counterpulsation during cardiogenic shock and high risk percutaneous coronary intervention (PCI) [15-17]. Two of these reported IABP-induced improvement in microvascular flow whereas the other did not. Therefore our understanding of microvascular perfusion during IABP-support remains based on limited and conflicting data. This paucity of data is even more apparent in deciding when to best withdraw IABP-support. No previous study has addressed this issue. To examine the influence of IABP-support on microcirculation of recovered patients, we studied patients deemed ready for discontinuing IABP-support as judged by their treating physicians. We used SDF-imaging to test our hypothesis that microvascular flow is unaltered with or without IABP-support in this clinical setting.

## Materials and methods

The local institutional review board approved the study protocol. Since the study was observational and given the non-invasive nature of SDF-imaging, the need for a written informed consent was waived in accordance with the national Law on Experiments with Humans. The study was performed at the intensive care unit (ICU) of a large teaching hospital between April 2007 and October 2008.

We included adult patients that had an IABP in place. Patients were only included if and when the responsible ICU physician had made the decision that the subjects were clinically ready for discontinuation of IABP-support. We excluded patients that showed signs of sepsis (suspected or proven systemic infection with ≥2 severe inflammatory response syndrome (SIRS) criteria: tachycardia >90 beats/minute and/or tachypnoea >20 breaths/minute or arterial pCO_2 _< 32 mmHg/4,2kPa and/or body temperature >38°C or <36°C and/or WBC count >12,000 cells/mm^3 ^or <4,000 cells/mm^3 ^or >10% immature cells). Disruption or laceration of the oral floor mucosa was an exclusion criterion because this would interfere with microcirculatory imaging. As per clinical routine, all patients underwent continuous invasive monitoring of arterial and central venous blood pressure and some patients had a surgically placed pulmonary artery catheter.

A Datascope^® ^CS300 intra aortic balloon pump system (Datascope Corporation, Mahwah, NJ, USA) was used in all studied patients. The IABP system was set automatically, using the electrocardiogram for optimal timing so that inflation and deflation occurred at the dicrotic notch and immediately before systolic upstroke, respectively. Optimal balloon size was chosen depending on patient height before insertion. Routine chest X-rays were examined to define correct intra-aortic placement of the IABP balloon, 2 to 3 cm distal to the origin of the left subclavian artery. All hemodynamic parameters were recorded continuously by our patient data management system.

The decision to discontinue IABP-support was a clinical one and left completely at the discretion of the ICU-team. In most cases, this included a weaning trial in which the IABP-assist ratio was lowered step by step over several hours. Possible changes in routinely measured macrocirculatory and laboratory parameters were observed during this process.

**Figure 1:**
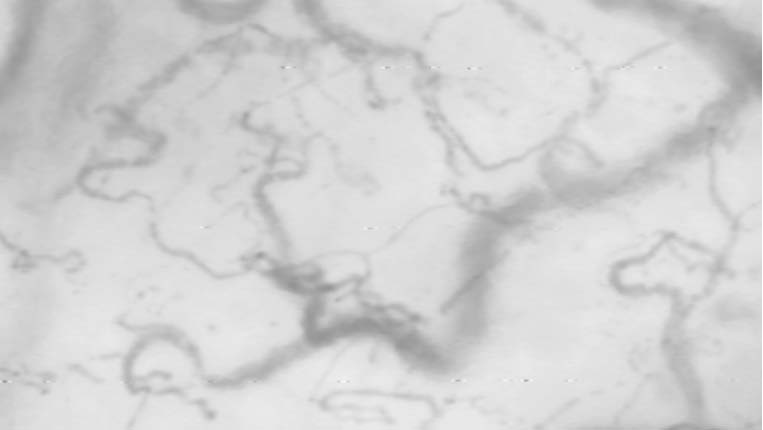
**A screenshot from a typical example of the human sublingual microcirculation using SDF imaging**.

Microcirculatory measurements were performed using SDF imaging, which has been described in detail elsewhere [7]. In brief, it consists of a handheld video microscope that emits stroboscopic green light (530 nm) from an outer ring at the tip of the probe. This light is absorbed by haemoglobin. A negative image of moving red blood cells is sent back through the isolated optical core of the probe toward a charge-coupled device (CCD) camera. SDF imaging has been shown to provide a higher imaging quality with more detail and less motion blur than its predecessor Orthogonal Polarization Spectral (Ops) imaging [7]. A typical example of a SDF-image is shown in Figure 1.

Within two hours after the decision to discontinue IABP-support had been made, we performed SDF imaging at two points in time. First, the IABP device was set to a 1:1 assist ratio, if this was not already the selected mode. After 10 minutes, to allow for a new steady state to occur, the first series of microvascular recordings was made. Next, the IABP device was temporarily stopped. After another 10 minutes, we recorded a second series of SDF video clips. At both measuring points, venous and arterial blood gas analyses were collected. In patients with a pulmonary artery catheter mixed venous saturation (SvO_2_) was determined, otherwise central venous saturation (ScvO_2_) was measured. After the procedure the IABP device was switched back on at the pre-measurement settings. During the procedure dosages of continuous intravenous drugs were recorded and no dosing adjustments were made.

In a recent round table conference, international experts reached consensus on how to best evaluate the microcirculation using OPS and SDF imaging [18]. We implemented all recommendations given in this conference. Video clips were immediately saved as digital AVI-DV files to a hard drive of a personal computer using an analogue-to-digital converter (Canopus, Kobe, Japan) and the freeware program WinDV [19]. We used 5× optical magnification, producing images representing approximately 940 × 750 mm^2 ^of tissue surface. Per measuring point, clips at three sublingual sites yielding at least 20 seconds of stable video per site were recorded. Special care was taken to avoid pressure artefacts, adhering to the standard operating procedure previously described by Trzeciak *et al. *[20] and recommended in the round table conference [18]. In brief, secretions were removed with gauze, and, after obtaining good imaging focus, the probe was pulled back gently until contact was lost and then advanced again slowly to the point at which contact was regained. The authors paid special attention to the larger vessels at the time of recording because alterations in their flow with probe manipulation may indicate pressure artefacts.

One video file was recorded for each location at each measuring point. These were stored under a random number. At a later time, these were analyzed by one of the authors (PWGE) using the AVA 3.0 software program (Microvision Medical, Amsterdam, The Netherlands). According to the recommendations, microvascular flow index (MFI), perfused vessel density (PVD), proportion of perfused vessels (PPVs), and indices of heterogeneity were determined for every patient at both time points. All have been validated previously [20-22]. As published recently, each score was determined for both large and small microvessels, with a cut-off diameter of 20 μm [23]. In addition, the authors defined large-type vessels that split into other vessels as arterioles. Other large vessels were defined as venules.

For PPV and PVD, vessel density was calculated as the number of vessels crossing three horizontal and three vertical equidistant lines spanning the screen divided by the total length of the lines. Perfusion at each crossing was then scored semi-quantitatively by the eye as follows: 0 = no flow (no flow present for the entire duration of the clip), 1 = intermittent flow (flow present <50% of the duration of the clip), 2 = sluggish flow (flow present >50% but <100% of the duration of the clip or very slow flow for the entire duration of the clip), and 3 = continuous flow (flow present for the entire duration of the clip). PVD was then calculated as the number of crossings with flow scores greater than 1. PPV was calculated as the proportion of crossings with flow scores greater than 1 divided by the total number of crossings. For each measuring point and each patient, the scores for PPV and PVD were averaged. PVD is expressed in n/mm, whereas PPV is expressed as a percentage. Intra-observer variability ranges between 2.5% and 4.7% for PPV and between 0.9% and 4.5% for PPV. The inter-observer variability is slightly higher: between 3.0% and 6.2% and between 4.1% and 10%, respectively [21].

MFI was based on the determination of the predominant type of flow in four quadrants adhering to the same scoring system. MFI is the sum of these flow scores divided by the number of quadrants in which the vessel type is visible. The intra-observer agreement of MFI is about 85% (kappa score = 0.78) and inter-observer agreement about 90% (kappa score = 0.85) [22]. For each measuring point and each patient, the scores for MFI were averaged.

Heterogeneity was assessed in two different ways. For PVD, the coefficient of variation was determined. For MFI, the authors assessed heterogeneity in each patient by subtracting the lowest from the highest quadrant MFI and dividing the result by the mean MFI [20,21].

We used Wilcoxon matched pairs tests for MFI and paired *t *tests for other data. We used Spearman tests to detect correlation between global and microvascular parameters. Results are reported as median and interquartile ranges (IQR) for MFI and as the mean ± standard deviation (SD) for other parameters, unless indicated otherwise. The study was powered to detect a minimum of 15% difference in small-vessel PVD after switching off the IABP with α = 0.05 and 1-β = 0.80. Based on previous studies by others and us [8,10,21] this showed the need for inclusion of 14 patients.

## Results

### Patients

We included 15 patients. Baseline characteristics of the study population, including risk factors for cardiovascular disease and continuous intravenous dosage of vasoactive drugs, are shown in Table 1. All participants were admitted at the intensive care unit of St. Antonius Hospital, Nieuwegein, The Netherlands.

**Table 1:** Patient characteristics and drugs

	Mean ± SD or n (%)
Number of patients	15
Age (years)	65.7 ± 11.8
Male	12 (80)
Length (cm)	175 ± 11
Weight (kg)	81 ± 14
Hemoglobin level (μmol)	6.2 ± 0.7
Hematocrit (%)	30 ± 3.5
Arterial blood lactate (mmol/l)*	1.2 ± 0.4
Temperature (°C)	36.9 ± 1.0
APACHE II score	14.5 ± 5.3
History of vascular disease	5 (33)
Hypertension	6 (40)
Diabetes Mellitus	3 (20)
Chronic renal insufficiency	1 (7)
Dopamine (mg/h), nine patients	17.7 ± 8.7
Norepinephrine (mg/h), five patients	0.26 ± 0.2
Dobutamine (mg/h), two patients	15.0 ± 7.1
Nitroglycerine (mg/h), six patients	1.0 ± 0.0
Artificial ventilation	11 (73)

Values are the number of patients, with percentages in parentheses. Catecholamine and vasopressors doses represent those used during SDF imaging. Other figures are mean ± standard deviation. *, When measured, 11 patients; APACHE II, acute physiology and chronic health evaluation scoring system II; SDF, sidestream dark field imaging

Mean APACHE (acute physiology and chronic health evaluation scoring system) II score 24 hours after admission at the ICU was 14.5 ± 5.3. Indications for IABP placement were urgent coronary artery bypass grafting due to unstable angina pectoris with severe coronary disease (*n *= 6, 40.0%), cardiogenic shock due to myocardial infarction without heart surgery (*n *= 2, 13.3%), cardiogenic shock after heart surgery (*n *= 2, 13.3%), due to acute prosthetic aortic valve displacement after surgery (*n *= 1, 6.7%) or after cardiac arrest with or without percutaneous coronary intervention (*n *= 4, 26.6%). Mean duration of IABP-therapy at the time of measurement was three days (range one to five days). During the experiment, six patients had a pulmonary artery catheter in place and 11 patients were mechanically ventilated.

### Systemic hemodynamic data

Table 2 depicts the differences in global hemodynamic parameters between the two points of interest. The institution of IABP-support significantly increased mean arterial pressure (MAP). However, recorded diastolic blood pressure was significantly lower. It is important to point out that this represents the lowest pressure recorded during the cardiac cycle, which is the purpose of balloon counterpulsation. After switching off IABP-support no differences in venous oxygen saturation occurred (ScvO_2_/SvO_2_; 70.9% ± 7.2 vs. 71.4% ± 7.5; *P *= 0.897).

**Table 2:** Global hemodynamic data

	Assist ratio 1:1	No assist	Difference	95% CI lower boundary	95% CI upper boundary	*P-*value
Heart rate (beats/minute)	83.4(12.1)	85.1(12.8)	-1.7	-4.45	0.98	0.193
ABP systolic (mmHg)	115.3 (16.0)	110.3 (15.0)	5.0	-2.18	12.18	0.157
ABP mean (mmHg)	73.7 (8.2)	70.7 (10.0)	2.9	0.03	5.83	0.048
ABP diastolic (mmHg)	42.6 (10.0)	53.3 (8.8)	-10.7	-15.20	-6.10	0.0002
CVP (mmHg)	12.5 (6.0)	13.0 (6.6)	-0.5	-1.72	0.78	0.437
ScvO_2_/SvO_2 _(%)	70.9 (7.2)	71.4 (7.5)	-0.5	-1.48	1.31	0.897
SpO_2 _(%)	96.4 (2.3)	96.5 (2.1)	-0.1	-0.91	0.65	0.719
PAP systolic (mmHg)	46.0 (8.3)	46.3 (6.8)	-0.3	-2.78	2.12	0.741
PAP mean (mmHg)	28.8 (4.2)	28.5 (4.3)	0.3	-0.75	1.41	0.465
PAP diastolic (mmHg)	22.7 (8.1)	22.5 (7.8)	0.2	-0.62	0.95	0.61

Data are presented as mean ± standard deviation in parentheses. ABP, mean arterial pressure; CI, confidence interval; CVP, central venous pressure; PAP, pulmonary artery pressure; ScvO_2_/ScvO_2_, central venous/mixed venous oxygen saturation; SpO_2_, peripheral oxygen saturation.

### Microcirculation

**Figure 2:**
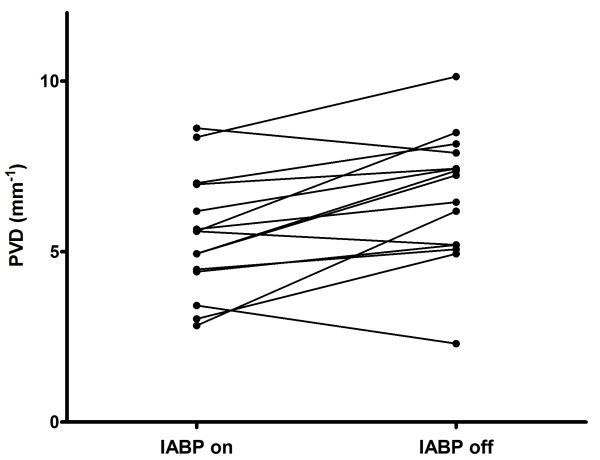
**The effect of IABP on perfused vessel density (PVD) for vessels <20 μm**. The perfused vessel density increased significantly, 5.47 ± 1.76 to 6.63 ± 1.90 (*P *= 0.0039), 10 minutes after IABP switch-off.

The authors successfully obtained high-quality images in each patient. In total 90 video clips were recorded. Results are shown in Table 3. PVD of small vessels (< 20 μm) was significantly lower during IABP-support; 5.47 ± 1.76 vs. 6.63 ± 1.90; *P *= 0.0039 (Figure 2). Other microcirculatory parameters were not significantly altered.

**Table 3:** Microcirculation

	Assist ratio 1:1	No assist	Difference	95% CI lower boundary	95% CI upper boundary	*P-*value
PVD vessels <20 μm (mm^-1^)	5.47 (1.76)	6.63 (1.90)	-1,16	-1.89	-0.44	0.0039
PPV vessels <20 μm (mm^-1^)	88.4 (14.1)	88.8 (16.4)	-0,43	-6.49	5.61	0.879
MFI vessels <20 μm	2.75 [2.0 to 3.0]	3.0 [2.58 to 3.0]	-0.25	NA	NA	0.422
HI-PVD vessels <20 μm	0.38 (0.23)	0.40 (0.37)	-0,02	-0.19	0.15	0.834
HI-MFI vessels <20 μm	0.68 (0.68)	0.57 (0.87)	0,11	-0.27	0.49	0.547
PVD vessels >20 μm (mm^-1^)	1.34 (0.88)	1.64 (0.63)	-0,31	-0.83	0.21	0.222
PPV vessels >20 μm (mm^-1^)	95.5 (9.6)	96.9 (7.7)	-1,38	-4.94	2.18	0.420
MFI vessels >20 μm	3.0 [2.5 to 3.0]	3.0 [2.78 to 3.0]	0	NA	NA	0.109
HI-PVD vessels >20 μm	0.59 (0.25)	0.50 (0.25)	0,08	-0.08	0.25	0.299
HI-MFI vessels >20 μm	0.44 (0.58)	0.18 (0.29)	0,26	-0.05	0.57	0.096

MFI is reported as median with interquartile ranges in square brackets. Other microcirculatory parameters are mean values ± standard deviation in parentheses. CI, confidence interval; HI, heterogeneity index; MFI, microvascular flow index; NA, not applicable; PPV, percentage of perfused vessels; PVD, perfused vessel density.

The relationship between changes in mean arterial pressure, diastolic arterial pressure and S(c)vO_2 _versus changes in PVD in individual patients comparing maximal support vs. no support was determined. Spearman tests did not show a statistically significant correlation for any of these global hemodynamic parameters (r = -0.1; *P *= 0.71, r = 0.1; *P *= 0.72, r = 0.1; *P *= 0.69 respectively). In addition, no correlation was found between small vessel PVD and APACHE II score (r = 0.04; *P *= 0.88).

## Discussion

To the best of our knowledge, this is the first study to report on the microvascular effects of IABP switch-off in recovered patients deemed ready for IABP removal. Our most prominent finding is that cessation of IABP-support resulted in a significant and paradoxical increase in small-vessel PVD, independent from initial disease severity. This is in contrast with the loss of IABP-induced raise of mean arterial pressure in these patients [1-3] and, perhaps more importantly, in contrast with SvO_2 _or ScvO_2 _values, which remained unchanged after IABP switch-off. Therefore, this study adds to the large body of evidence that global hemodynamic parameters and venous oximetry do not necessarily reflect microvascular perfusion [4,9,10,21,22]. It is the first time that such a discrepancy is demonstrated after withdrawal of IABP-support.

Three recent studies examined the effects of IABP on human microcirculation [15-17]. Jung *et al. *included 13 patients with cardiogenic shock after acute myocardial infarction. The authors recorded SDF video images before and shortly after the IABP-support was temporarily stopped. MFI of small and medium vessels (10 to 50 μm) was significantly higher in patients with IABP-support [15]. In contrast, den Uil *et al. *studied a heterogeneous group of 13 patients suffering from cardiogenic shock of variable severity and found no differences in perfused capillary density (PCD) and red blood cell velocity [16]. This was despite the fact that mean arterial pressure and cardiac index were significantly lower after the IABP-assist ratio was switched from 1:1 to 1:8. Finally, more recently, Jung *et al. *studied six patients immediately following high-risk PCI. MFI of both small and large vessels decreased significantly immediately after a short period of IABP-support discontinuation and returned to baseline after restarting therapy. Again, no correlation with global hemodynamic parameters was found [17].

Both groups chose not to fully incorporate recent recommendations regarding image acquisition or reporting standard microvascular flow parameters [18]. This hampers direct comparison with our study. More importantly, our population of recovered patients is markedly different. However, this does not dismiss our sharply contrasting finding of improved PVD after IABP switch-off. It is plausible that IABP has different microvascular effects at different stages of disease and recovery.

Although speculative, one possible explanation for the observed difference in microvascular perfusion could be a mechanical effect of IABP. The pressure decrease induced by balloon deflation during diastole could create a collapse of certain parts of capillary microcirculation. This effect is consistent with the concept of vascular waterfalls: the flow in front of a waterfall is not affected by its height. This analogy explains why sometimes global and regional blood flow ceases at positive arterial pressures well above venous pressure [24,25]. Interestingly, an IABP-induced transient blood flow reversal in basal cerebral arteries has been reported previously [26,27], which is consistent with this theory. However, correlation between the difference in diastolic pressure and the difference in PVD between the study time points did not reach statistical significance. Conversely, this may not invalidate our hypothesis, as microvascular de-recruitment is probably an all or nothing stochastic process.

There are several important limitations to our study. First, we did not routinely measure cardiac output. However, this is highly representative of current practice. In addition, it may be reasonable to assume that cardiac output remained constant as no changes in venous oximetry and heart rate were observed and no drug dosing adjustments were made during the study period. Interestingly, if IABP would have increased cardiac output in our patients, as it has been reported to do so in other clinical situations, this would perhaps even strengthen our findings. This would mean that despite increased cardiac output during IABP-support, microvascular flow would be relatively impaired as compared to no IABP-support.

Second, we chose the sublingual site for its proximity to the brain, ease of access and its philogenetic relationship to the gut. There is conflicting data on whether sublingual microvascular perfusion represents other vascular beds [28,29]. Interestingly, a recent animal study shows that in the setting of resuscitation, cerebral microcirculation is relatively protected as compared to sublingual microvascular perfusion [30]. If applicable to the setting studied by us, this would imply that cerebral microvascular perfusion improves to a greater extent after IABP switch-off. Third, the fact that our findings could possibly be explained by temporal changes in microcirculation instead of IABP cannot be ruled out because we did not perform a final measurement after reinstituting IABP-support. Finally, but perhaps most important, a heterogeneous group was studied in which a minority initially had cardiogenic shock. However, no correlation of initial disease severity (APACHE II score) and microcirculatory perfusion at the time of measurement could be found. Further, there were no differences in microcirculatory perfusion between patients with initial shock and those without shock. The absence of correlation between microvascular flow and APACHE II and shock state may not be surprising as patients were only studied during a phase in which they were deemed ready for discontinuation of IABP-support.

Given these limitations, our findings and their clinical consequences should be interpreted with caution. In addition, while we have shown that IABP may impair tissue perfusion in hemodynamically recovered patients, we did not observe signs of cell ischemia (for example, lactate acidosis) nor signs of progressive organ dysfunction during IABP-therapy on the day measurements were performed. In addition PVD values during IABP-support are similar to those found in healthy controls by De Backer [8] and to those found after routine cardiac surgery by us [10].

However, given the current controversy on the evidence of IABP-support and therefore its indication [31], coupled with complication risks [32], it may be prudent not to ignore our results. Perhaps the best strategy is to optimize the duration of support and our findings could possibly be the consequence of the fact that our patients may have been too long IABP-treated. This could explain our findings. The role of microvascular imaging to achieve this goal merits further study.

## Conclusions

The results of this study show that cessation of IABP-support resulted in a paradoxical increase of microvascular flow in small vessels at a time clinicians deemed IABP was no longer necessary. These changes are independent from global hemodynamic parameters and oxygen derived variables.

Clinicians that routinely rely on these parameters for their decision-making should again be reminded that these do not necessarily reflect microvascular perfusion in a wide range of clinical settings including that of cessation of IABP-support.

## Key messages

• Discontinuing IABP-therapy increases sublingual microvascular perfusion of small vessels in patients deemed clinically ready for ceasing IABP-support.

• Global hemodynamic parameters and venous oximetry do not correlate with and may therefore be unreliable for predicting adequacy of microvascular perfusion in this clinical setting.

## Abbreviations

APACHE: acute physiology and chronic health evaluation scoring system; CCD: charge-coupled device; IABP: intra aortic balloon pump; MAP: mean arterial pressure; MFI: microvascular flow index; OPS: orthogonal polarization spectral imaging; PCD: perfused capillary density; PCI: percutaneous coronary intervention; PPV: proportion of perfused vessels; PVD: perfused vessel density; ScvO_2_: central venous oxygen saturation; SD: standard deviation; SDF: sidestream dark field imaging; SvO_2_: mixed venous oxygen saturation.

## Competing interests

CI is chief scientific officer of Microvision Medical. Microvision Medical is a university-based company dedicated to the development of optical spectroscopic tools for study of the microcirculation and tissue oxygenation, in which context CI holds patents and shares. All other authors declare they have no competing interests.

## Authors' contributions

LM participated in the design of the study, performed data acquisition and drafted the manuscript. PGWE conceived the study, performed data processing and drafting of the manuscript. AO participated in data acquisition. ED, MI and CI have participated in the design of the study and critically revising of the manuscript.
